# CAP1, a target of miR‐144/451, negatively regulates erythroid differentiation and enucleation

**DOI:** 10.1111/jcmm.16067

**Published:** 2021-01-26

**Authors:** Xiaoli Huang, Ruihua Chao, Yanyang Zhang, Pengxiang Wang, Xueping Gong, Dongli Liang, Yuan Wang

**Affiliations:** ^1^ Shanghai Key Laboratory of Regulatory Biology Institute of Biomedical Sciences and School of Life Sciences East China Normal University Shanghai China; ^2^ Department of Animal Sciences College of Agriculture and Natural Resources Michigan State University East Lansing MI USA

**Keywords:** actin filament remodelling, CAP1, enucleation, erythropoiesis, *miR‐144/451*

## Abstract

The exact molecular mechanism underlying erythroblast enucleation has been a fundamental biological question for decades. In this study, we found that *miR‐144/451* critically regulated erythroid differentiation and enucleation. We further identified CAP1, a G‐actin‐binding protein, as a direct target of *miR‐144/451* in these processes. During terminal erythropoiesis, CAP1 expression declines along with gradually increased *miR‐144/451 levels*. Enforced CAP1 up‐regulation inhibits the formation of contractile actin rings in erythroblasts and prevents their terminal differentiation and enucleation. Our findings reveal a negative regulatory role of CAP1 in *miR‐144/451*‐mediated erythropoiesis and thus shed light on how microRNAs fine‐tune terminal erythroid development through regulating actin dynamics.

## 1 INTRODUCTION

Definitive erythropoiesis is an essential biological process that sustains the consumption and regeneration of end‐stage erythroid cells through one's life span. During this process, erythroblasts from hematopoietic stem cells become enucleated reticulocytes at foetal liver during the embryonic stage or at bone marrow after birth.^1^, ^2^, ^3^ The first committed progenitor in definitive erythropoiesis is the burst forming unit‐erythroid, which differentiates into colony‐forming unit‐erythroid (CFU‐E) in response to erythropoietin (EPO).^4^, ^5^, ^6^, ^7^ The CFU‐E progenitors further undergo sequential developmental stages of proerythroblasts, basophilic erythroblasts and polychromatic erythroblasts and eventually become orthochromatic erythroblasts.^8^ Substantial changes occur during erythroid differentiation, including nuclear condensation, decreased cell size and haemoglobinization.^3^, ^8^ Therefore, erythroid differentiation is typically accompanied by significant increases of haem biosynthetic enzymes and synthesis of α/β‐globins, as well as changes in membrane components and cytoskeleton.^3^, ^8^, ^9^, ^10^ Enucleation starts in orthochromatic erythroblasts, followed by an expulsion of organelles in reticulocytes.^8^ These events are strictly regulated to ensure the final proper production of functional erythroid cells. For example, Rac GTPases regulate the formation of contractile actin rings (CAR) on the plasma membrane of erythroblasts, which in turn contribute to their enucleation.^11^

MicroRNAs (miRNAs) are critical regulators of erythropoiesis.^12^, ^13^, ^14^ Specially, high expression of *miR‐144* and *miR‐451* from a bicistronic miRNA locus has been observed in erythroid lineages of various species, including human ^15^, ^16^ and mouse.^15^, ^17^, ^18^ The *miR‐144/451* appears to be a positive regulator of erythroid maturation.^19^ Severe anaemia develops in *miR‐144/451* knockout mice with erythropoiesis deficiency, including apoptosis of erythroblasts,^20^ reduction in erythroid number and increased distribution width of red blood cells.^19^ In addition, the anaemia upon *miR‐144/451* deletion deteriorates under oxidative stress.^21^, ^22^ Consistent with this observation, several downstream targets of *miR‐144/451* have been identified, including *NRF2*,^23^ *Ywhaz* ^21^, ^22^ and *Cab39*.^20^ All these *miR‐144/451* targets participate in cellular reductive/oxidative processes and protect erythrocytes from oxidant damage.^20^, ^21^, ^22^, ^23^ However, it remains elusive how abnormal morphologic phenotypes, such as the increased distribution width of red blood cells and blocked enucleation, occur in *miR‐144/451^−/−^* erythrocytes, suggesting there are undefined targets of *miR‐144/451* during definitive erythropoiesis.

Cyclase‐associated proteins (CAPs) are highly conserved actin‐binding proteins in eukaryotic organisms.^24^, ^25^, ^26^, ^27^ *Srv2*, the CAP homologue, first identified from yeast, is a binding partner of adenylyl cyclase and an effector of *Ras* during nutritional signalling.^25^, ^28^ Mammals have two CAP paralogues, CAP1 and CAP2.^29^ In mice, CAP1 is expressed in most non‐muscle cell types, whereas CAP2 is primarily restricted to certain brain regions and striated muscles.^30^ CAP1 was reported to be a binding protein of globular actin (G‐actin, monomeric).^31^ In a head‐to‐tail fashion, G‐actin polymerizes to form a helical F‐actin filament with a defined polarity, in which F‐actin filaments elongate at one end whereas simultaneously shrink at the other end by releasing monomeric G‐actin.^32^ In general, proteins interacting with F‐actin contribute to the assembly of actin filaments and the construction of F‐actin network, whereas G‐actin‐binding proteins serve to sequester monomeric actin and modulate the availability of un‐polymerized G‐actin.^24^ The actin cytoskeletal dynamics are crucial for many cellular processes including erythroblast enucleation and proper assembly of red blood cell membrane, two key steps of terminal erythropoiesis in mammals.^33^, ^34^ As a G‐actin‐binding protein, CAP1 was reported to sequester actin monomers to prevent their polymerization ^31^ and to stimulate nucleotide exchange of ATP onto ADP‐bound G‐actin, a rate‐limiting step in regenerating polymerizable G‐actin.^35^ In addition, CAP1 was found to accelerate the depolymerization of F‐actin by coordinating with an actin‐cofilin complex.^36^, ^37^ It however remained undefined what the functions of CAP1 are in erythroid enucleation and membrane assembly. Nor is clear how CAP1 is regulated in definitive erythropoiesis. In the current study, we revealed critical roles of CAP1 in maintaining cellular actin dynamics during erythroid development. We identified CAP1 as a direct target of *miR‐451* and found that CAP1 down‐regulation was essential for erythroid differentiation and enucleation.

## 2 MATERIALS AND METHODS

### 2.1 Mouse breeding and foetal liver erythroid differentiation

Female C57BL/6 mice at 12.5 and 14.5 days of gestation were sacrificed using cervical dislocation and foetal livers were collected from embryos. Purification and in vitro differentiation of foetal liver erythroblast precursors were performed according to published protocol^11^ with minor modifications. CD71+/Ter119− foetal cells were sorted by flow cytometry Aria II (BD Biosciences) and seeded in fibronectin‐coated wells at a cell density of 1 × 10^5^/mL. At day 1, purified cells were cultured in IMDM (Thermo Fisher Scientific) containing 1% detoxified BSA (Sigma‐Aldrich), 15% FBS (Thermo Fisher Scientific), 200 g/mL holo‐transferrin (Sigma‐Aldrich), 2 U/mL EPO (Amgen), 2 mmol/L l‐glutamine (Thermo Fisher Scientific), 10 g/mL recombinant insulin (Sigma‐Aldrich) and 10^−4^ mol/L β‐mercaptoethanol (Sigma‐Aldrich). At day 2, the medium was replaced with erythroid‐differentiation medium (IMDM containing 20% FBS, 10^−4^ mol/L β‐mercaptoethanol and 2 mmol/L l‐glutamine). Foetal liver erythroblast transfection, 75 pmol siRNA was transfected into 1‐2 × 10^6^ cells with Lipofectamine™ 3000 Transfection Reagent (Invitrogen) following the manufacturer's instruction. For introduction of plasmids containing transgenes, 1‐5 μg of plasmid DNA per 5‐10 × 10^5^ cells was electroporated into the sorted CD71+/Ter119− cells with P3 primary cell 4D nucleofector™ X Kit L (Lonza) using program CD34+ of 4D‐Nucleofector™ Core Unit System (Lonza). All animal experimental procedures were conducted in accordance with the local Animal Welfare Act and Public Health Service Policy with approval from the Committee of Animal Experimental Ethics at East China Normal University (Ref #:M20170320).

### 2.2 Cell culture and DMSO‐induced erythroid differentiation in vitro

Murine erythroleukemia (MEL) cells ^38^ were cultured in RPMI‐1640 medium (Thermo Fisher Scientific) supplemented with 10% FBS and 100 U/mL of penicillin/streptomycin (Thermo Fisher Scientific). Erythroid differentiation of MEL cells was induced by adding dimethyl sulfoxide (DMSO; Sigma‐Aldrich) to 2% as the final concentration in culture media.^39^ Differentiated cells were collected at designated time points after DMSO treatment. 293T cells were cultured in DMEM with 10% FBS.

### 2.3 Benzidine staining

Benzidine staining was performed following the published protocols.^40^ Briefly, a 0.2% solution of benzidine (Sigma‐Aldrich) in 3% acetic acid was freshly prepared. Prior to use, hydrogen peroxide was added to 0.3% as the final concentration. MEL cells were first smeared on glass slides, air‐dried, stained in the above described solution for 10 minutes (shielded from light) and finally fixed in 100% Methanol for 10 minutes. Images were taken with a Leica microscope.

### 2.4 May‐Grunwald‐Giemsa staining

May‐Grunwald‐Giemsa staining was performed following the published protocols.^41^ Briefly, cells were first smeared onto glass slides, air‐dried, fixed in 100% Methanol for 3‐10 minutes and then went through 2‐step staining: first with 50% (v/v) May‐Grunwald (Sigma, CAS NO. 63590) in phosphate‐buffered saline (pH 6.8) for 3‐5 minutes, then with 10% Giemsa (Sigma, CAS NO. 48900) for 10‐30 minutes and washed with running water for 1‐3 minutes. Images of air‐dried slides were taken under a Leica microscope.

### 2.5 Contractile actin ring (CAR) staining

For CAR staining, MEL cells were harvested in PBS. Cell pellets were fixed in 100 µL PBS with 0.5% acrolein for 5 minutes, and cell concentration was adjusted to approximately 5 × 10^6^ cells/mL. Cells (~100 µL) were applied to poly‐l‐lysine‐coated slides and dried at room temperature. The slides were rinsed three times in PBS to remove unbound cells. Cells were permeabilized in PBS containing 0.05% Triton X‐100 for 10 seconds, followed by three washes in PBS, then incubated in blocking buffer (PBS containing 0.5 mmol/L glycine, 0.2% fish skin gelatin and 0.05% sodium azide) for 1 hour and incubated with 1 U/mL Alexa Fluor 568–phalloidin (Thermo Fisher Scientific) for 1 hour. Slides were washed three times in blocking buffer followed by DAPI staining for 15 minutes before images were taken with a Leica fluorescence microscope.

### 2.6 Flow cytometry analysis

Flow cytometry analyses of mouse foetal erythroblasts with CD71 and Ter119 were performed as previously described.^11^, ^42^ For enucleation analysis, cells were stained with 10 µg/mL Hoechst 33342 (Sigma–Aldrich), together with an APC‐Ter119 antibody, for 30 minutes at 4°C. Flow cytometry analyses and sorting were performed respectively on Calibur or Fortessa analyzers, and an AriaII cell sorter (BD Biosciences). Cell size changes during differentiation were measured with FSC‐A using flow cytometry. Anti‐mouse PE‐CD71, APC‐Ter119 antibodies and Annexin V Early Apoptosis Detection Kit (Cat#: 553786) were purchased from BD Biosciences.

### 2.7 Plasmid construction

Mouse *Cap1* cDNA was cloned by PCR and inserted into a lentiviral *pll3.7‐EF1α‐MCS‐IRES‐hygro* vector between *EcoRI/NotI* sites. To overexpress *pri‐miR‐144/451*, a DNA fragment containing the *pri‐miR‐144/451* cluster was PCR‐amplified from mouse genomic DNA and then inserted into a *MSCV‐MCS‐IRES‐hygro* vector between *EcoRI/XhoI* sites. Three *Cap1*‐specific shRNAs and a scramble shRNA control were designed and cloned into a *Plko.1‐U6‐shRNA‐puro* vector, respectively. Sequences of oligonucleotides and primers in this study are provided in the Table S1.

### 2.8 Quantitative real‐time PCR

Total RNAs were extracted with Trizol (Thermo Fisher Scientific), and cDNAs were synthesized using a PrimeScript^®^ RT reagent Kit (TaKaRa) following manufacturer's protocols, as previously described.^43^, ^44^ The primers used in this assay are provided in the Table S1.

### 2.9 Luciferase reporter assay

The 3′‐UTR fragment of mouse *Cap1* was cloned into a *pGL4‐basic* firefly luciferase reporter vector. Mutant 3′‐UTR fragment of mouse *Cap1* was generated by PCR‐based site‐directed mutagenesis. All constructs were verified by DNA sequencing. The reporter plasmids were co‐transfected into 293T cells with a Renilla luciferase expressing vector, and a *plvx‐ires‐zsgreen* plasmid containing the *pri‐miR‐144/451* driven by a CMV promoter. The firefly and Renilla luciferase activities were measured with the Dual‐Glo kit (Promega).

### 2.10 Western blotting

Total proteins were isolated using RIPA lysis buffer, subjected to SDS‐PAGE, transferred to PVDF membranes and incubated with primary antibodies. Quantification of target protein levels was performed using the ECL detection system and Quantity One software (Bio‐Rad). Antibodies used in this study: CAP1 (NBP1‐58320; Novus), β‐ACTIN (sc‐10731; Santa Cruz Biotech) and GAPDH (sc‐25778; Santa Cruz Biotech). The relative protein expression levels were determined by the density (grey mean value) of the protein bands with the ImageJ software and normalized to the respective loading control GAPDH/β‐ACTIN.

### 2.11 Statistical analysis

Data were presented as mean ± standard error (SEM). All experiments were performed independently for more than three times unless otherwise stated. Statistical analysis between group differences was performed with two‐tail unpaired Student's *t* test using Graph Prism software (version 5.0; GraphPad). A *P* value <.05 was considered significant (*P < .05, **P < .01, ***P < .001).

## 3 RESULTS

### 3.1 CAP1 is a direct target of *miR‐451*

**Figure 1:**
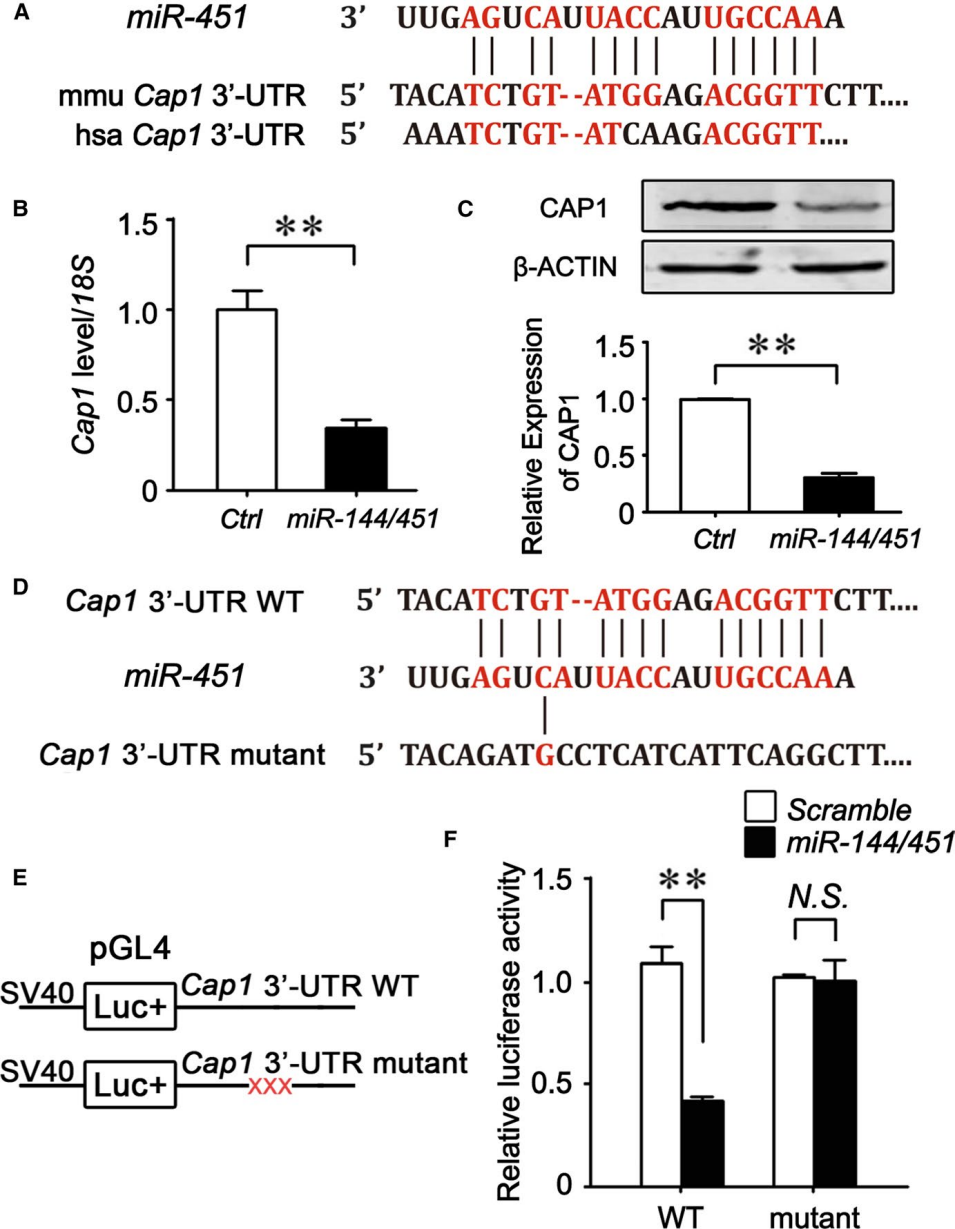
CAP1 is a direct target gene of *miR‐451*. A, Mouse *Cap1* 3′‐UTR contains *miR‐451*‐binding sites. B, *Cap1* mRNA levels in MEL cells were measured by real‐time RT‐PCR assays upon infection of an empty viral vector (Ctrl) or a vector with *pri‐miR‐144/451*. C, Western blotting and relative protein expression levels were determined by density of protein bands of CAP1 in MEL cells upon overexpressing *pri‐miR‐144/451* cDNA, compared with an empty vector control. D, The diagram of mutations introduced into the target sequences of mouse *Cap1* 3′‐UTR. E, The plasmid construction of dual‐luciferase reporter vector pGL4, containing either wild‐type or mutated *Cap1* 3′‐UTR from (D). F, Relative luciferase activities (Firefly vs Renilla luciferases) were determined in 293T cells at 48 h after transfection of an empty vector control or vectors with luciferase cDNA fused with wildtype or mutant *Cap*1 3’‐UTR (from E) in the absence or presence of *pri‐miR‐144/451*. B, C, F, The values are presented as the mean ± SEM (n ≥ 3; **P < .01; *NS*, no significance)

To understand the molecular mechanisms of how *miR‐144/451* impacts erythropoiesis, we first predicted their targets by conducting bioinformatics analyses through Targetscan, an online software. We identified CAP1 as a potential target of *miR‐451* (Figure 1A). Consistent with this finding, retroviral vector‐mediated overexpression of *pri‐miR‐144/451* in MEL cells reduced *Cap1* mRNA and protein levels to approximately 40% and 20%, respectively (Figure 1B,C), suggesting that *miR‐144/451* negatively regulates CAP1 expression.

To further investigate whether *miR‐451* directly targets *Cap1* mRNA, a dual‐luciferase reporter assay was conducted in 293T cells. In this assay, we constructed a firefly luciferase reporter, downstream of which was inserted with a wild‐type or mutant *Cap1* 3′‐UTR fragment containing mutations within the *miR‐451* seed targeting sequences (Figure 1D,E). We found that *pri‐miR‐144/451* significantly inhibited the activity of firefly luciferases linked with the wild‐type *Cap1* 3′‐UTR. By contrast, the inhibitory effect of *pri‐miR‐144/451* was lost in the vector containing *Cap1* 3′‐UTR with a mutated *miR‐451* seed target sequence (Figure 1F). Taken together, these results reveal that *Cap1* is a direct target gene of *miR‐451*.

### 3.2 CAP1 is down‐regulated during foetal erythroid differentiation

**Figure 2:**
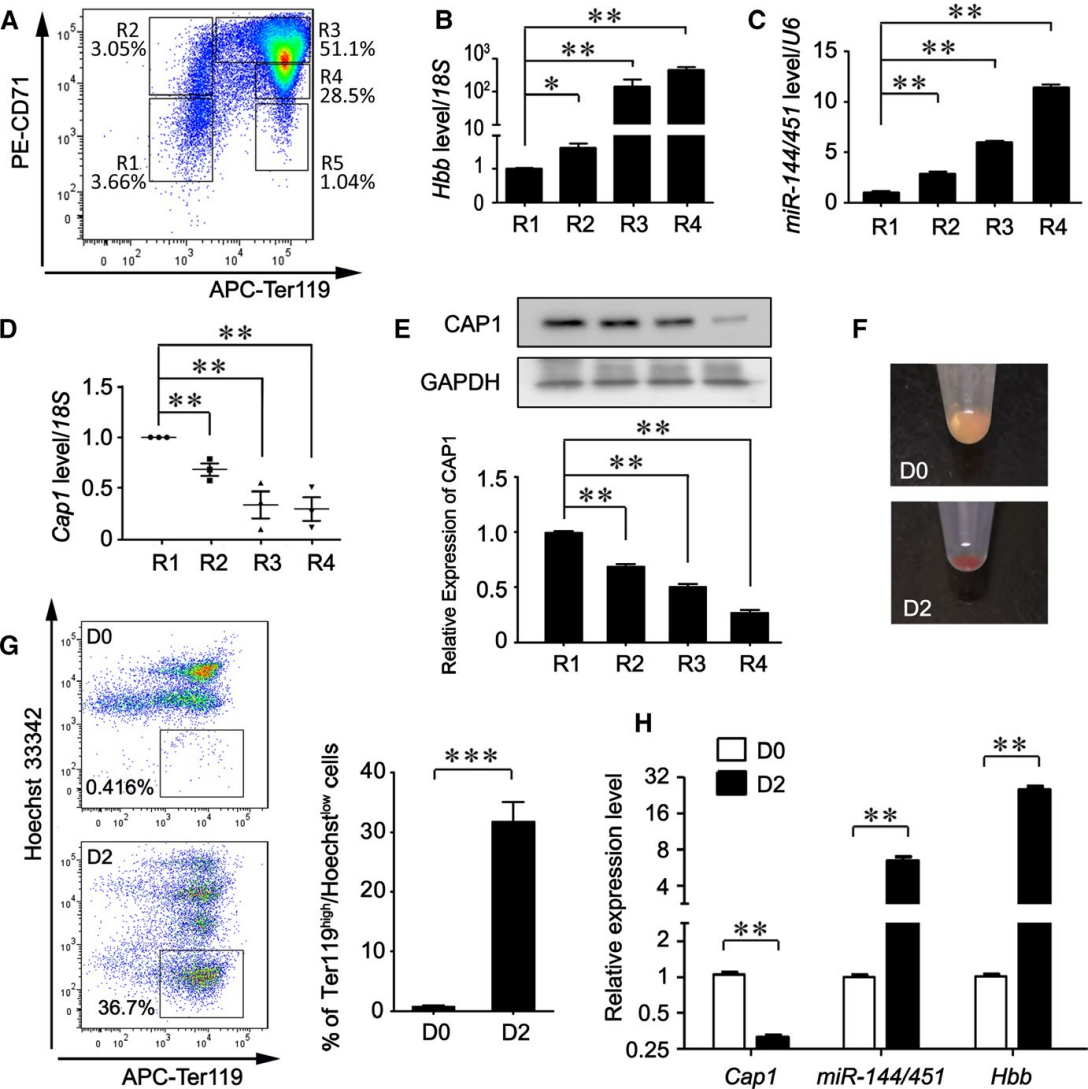
CAP1 is down‐regulated during terminal erythroid differentiation. A, A representative diagram of erythroid populations in E14.5 foetal livers, analysed by flow cytometry with antibodies against CD71 and Ter119. B‐D, R1 to R4 cell populations showed in (A) were sorted by flow cytometry. The mRNA levels of *β‐globin* (B), *pri‐miR‐144/451* (C) and *Cap1* (D) were then determined by real‐time RT‐PCR assays. *18S* (B & D) or *U6* (C) rRNAs were used as the internal reference controls. E, Western blotting analyses of CAP1 protein levels in R1 to R4 cell populations. F, Gross view of the cell pellets from D0 and D2 in vitro differentiated foetal liver erythroid cells. G, D0 and D2 in vitro cultured foetal liver cells were stained with APC‐Ter119 and Hoechst 33342. Representative plots of flow cytometry were shown on the left and averaged percentage of Ter119^high^/Hoechst^low^ population from 3 independent experiments on the right. H, Relative expression levels of *β‐globin*, *pri‐miR‐144/451* and *Cap1* from D0 and D2 in vitro cultured foetal liver cells. B‐D, E, G, H, All data are presented as mean ± SEM from at least three biological replicates (*P < .05; **P < .01; ***P < .001)

To study the role of CAP1 in erythroid development, we first examined its expression level at different stages of erythroid development. We utilized a foetal liver erythropoietic model because more than 90% of cell population from mouse foetal liver are of erythroid lineage.^11^ Five distinct erythroblast populations from mouse foetal livers at embryonic day 14.5 (E14.5) were identified with flow cytometry and designated as R1 to R5, according to their Ter119 and CD71 double‐staining patterns (Figure 2A): R1 (CD71^med^/Ter119^low^) is mainly comprised of primitive progenitor cells and proerythroblasts; R2 (CD71^high^/Ter119^low^) includes proerythroblasts and early basophilic erythroblasts; R3 (CD71^high^/Ter119^high^) is enriched with early and late basophilic erythroblasts; R4 (CD71^med^/Ter119^high^) contains chromatophilic and orthochromatophilic erythroblasts; and R5 (CD71^med^/Ter119^med^) represents late orthochromatophilic erythroblasts and reticulocytes.^11^ As shown in Figure 2B,C, both *β‐globin* (*Hbb*: *Hbb‐β1*&*β2*) and *pri‐miR‐144/451* were significantly up‐regulated all the way through R4 stage. By contrast, *Cap1* mRNA was promptly down‐regulated from R1 to R4 (Figure 2D). CAP1 protein showed a similar expression pattern (Figure 2E), supporting a negative reciprocal expression of CAP1 and *miR‐144/451* during erythroid differentiation.

We next examined *Cap1* expression levels during in vitro erythroid differentiation of CD71+/Ter119‐ foetal liver cells isolated from E12.5 mice (D0).^11^ After induced differentiation for 2 days (D2), we clearly observed red coloured cell pellets, indicating erythroid maturation with increased levels of iron‐containing haemoglobin (Figure 2F). In addition, double staining for Ter119 and a DNA dye Hoechst 33342 demonstrated a significant increase from 0.4% to 36.7% of the Ter119^high^Hoechst^low^ population (Figure 2G), which represents enucleated reticulocytes.^45^ Compared with D0 progenitors, *Cap1* expression dramatically decreased in D2 differentiated erythroid cells. This is in sharp contrast to the remarkable increased transcript levels of *β‐globin* (*Hbb*: *Hbb‐β1*&*β2*) and *pri‐miR‐144/451* (Figure 2H). Taken together, these results illustrate that CAP1 is down‐regulated both in vitro and in vivo along definitive erythroid differentiation, suggesting a vital role of CAP1 in this process.

### 3.3 CAP1 inhibits terminal erythroid differentiation of MEL cells

**Figure 3:**
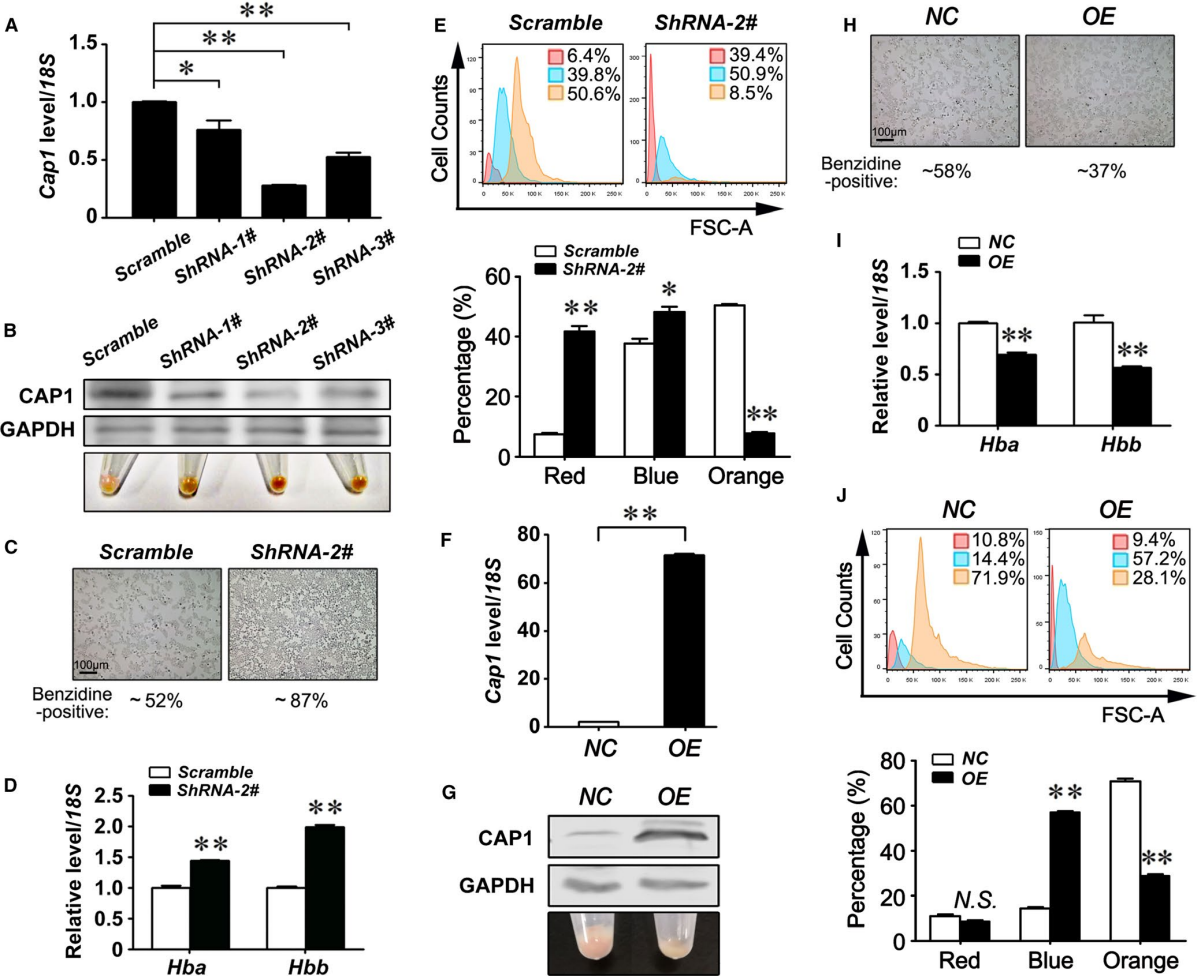
CAP1 inhibits terminal erythroid differentiation of MEL cells. A, *Cap1* mRNA levels in MEL at day 2 following the introduction of shRNAs against *Cap1* were determined by real‐time RT‐PCR. B, Gross view of the cell pellets and Western blotting analyses of CAP1 protein levels at day 4 during DMSO‐induced differentiation following shRNA knock‐down of CAP1 in MEL. C, Benzidine staining at day 4 after DMSO induction following CAP1 knock‐down in MEL. D, Haemoglobin mRNA levels in MEL were measured by real‐time RT‐PCR at day 4 during DMSO‐induced differentiation upon CAP1 knock‐down. E, The size distribution (FSC‐A) of MEL cells was analysed by flow cytometry at day 4 after DMSO‐induced differentiation following CAP1 knock‐down. Three groups of cells with various sizes were detected by flow cytometry. The percentage of each group was averaged from 3 independent experiments with representative plots shown above the graph. F, *Cap1* mRNA levels were determined by real‐time RT‐PCR at day 2 after introducing a *Cap1* cDNA into MEL. G, Western blot analysis of CAP1 protein levels at day 4 during DMSO‐induced differentiation upon CAP1 overexpression in MEL. A typical view of cell pellets after MEL differentiation upon CAP1 overexpression was shown. NC: empty vector control. H, Benzidine staining assays of MEL at day 4 during DMSO‐induced differentiation upon CAP1 overexpression. I, Haemoglobin mRNA levels in differentiated MEL were determined by real‐time RT‐PCR at day 4 after DMSO treatment with CAP1 overexpression, compared with an empty vector control. J, The size distribution (FSC‐A) of MEL cells was analysed by flow cytometry at day 4 after DMSO‐induced differentiation following CAP1 overexpression. The percentage of each group was averaged from three independent experiments with representative plots shown above the graph. A, D, E, F, I, J, The data are represented as the mean ± SEM (n ≥ 3; *P < .05; **P < .01; *NS*, no significance). OE, overexpression

To define the function of CAP1 in erythropoiesis, three *Cap1*‐specific shRNAs and a scramble shRNA control were introduced by lentiviral infection into MEL cells. MEL is an erythroleukemia line immortalized by the Friend virus complex.^46^ MEL cells are arrested before the proerythroblast stage in culture but can be induced by treatment of DMSO or other chemical agents to go through morphological and biochemical changes which mimic in vivo developmental events during erythroid differentiation.^47^ Real‐time RT‐PCR confirmed that *Cap1* expression was significantly inhibited by shRNA knock‐down in MEL cells, with shRNA #2 achieving the highest knock‐down efficiency at both mRNA and protein levels (Figure 3A,B). Strikingly, knock‐down of *Cap1* greatly facilitated MEL differentiation upon DMSO treatment (Figure 3B,C). At day 4 after DMSO treatment, the differentiated MEL cell pellets displayed a dark red colour in *Cap1* knock‐down groups, in sharp contrast to the scrambled control that showed only a tinge of redness (Figure 3B, lower panel). Benzidine is a chemical that forms a dark blue precipitate upon oxidation of the haem group in haemoglobin by hydrogen peroxide. It thus serves as a dye for the histochemical detection of differentiated red blood cells with high haemoglobin expression. We found that blue MEL cells were significantly increased from 52% to 87% upon *Cap1* knock‐down (Figure 3C and S1A). Consistent with these data, real‐time RT‐PCR assays showed that *Cap1* down‐regulation significantly promoted the expression of both *α‐globin* (*Hba*: *Hba‐α1* & *α2*) and *β‐globin* (*Hbb*: *Hbb‐β1* & *β2*) (Figure 3D).

Because erythroid cells become smaller during differentiation and enucleation, we next examined the size distribution of MEL cells upon DMSO induction. We were able to detect three MEL populations with different sizes by flow cytometry (Figure 3E): the large cells of erythroblasts (orange area with FSC‐A >50K), terminally differentiated small‐sized MEL cells (red area with FSC‐A <30K) and middle‐sized cells that were likely in the process of differentiation (blue area with FSC‐A peak spanning between 20K and 50K). We found significantly more small‐sized erythroid cells increasing from 46.2% to 90.3% upon CAP1 inhibition (red plus blue populations in Figure 3E), whereas the percentage of erythroblasts with large‐sized erythroblasts dropped from 50.6% to 8.5% (Figure 3E), supporting that CAP1 inhibition favours erythroid differentiation. We further performed May‐Grünwald Giemsa staining (MGG) staining to examine cell morphology.^41^ In this assay, acidic cytoplasm is stained light blue by methylene blue, whereas nuclei are generally stained purple/bluish violet due to interactions between eosin Y and Azure B‐DNA complex.^41^ We found that during DMSO‐induced differentiation, small MEL cells without purple nuclei were significantly increased in *Cap1* knock‐down group (Figure S1B), indicating that more MEL cells went through differentiation and enucleation when CAP1 was repressed.

Finally, we determined the influence of CAP1 overexpression on erythroid differentiation. Lentiviral *pll3.7‐Cap1‐hygro* vector was constructed and introduced into MEL cells. Real‐time RT‐PCR (Figure 3F) and Western blotting (Figure 3G, upper panel) assays confirmed the elevated CAP1 expression in the CAP1 overexpressing group, compared with an empty control. At day 4 after DMSO induction, the differentiated MEL cell pellets lost the tinge of red colour in the CAP1 overexpression group (Figure 3G, lower panel). Consistently, benzidine staining showed sharply decreased blue MEL cells upon *Cap1* overexpression (Figure 3H). The levels of *α‐globin* (*Hba*: *Hba‐α1* & *α2*) and *β‐globin* (*Hbb*: *Hbb‐β1* & *β2*) were correspondingly repressed by enforced CAP1 expression (Figure 3I). Interestingly, upon CAP1 overexpression, although the proportion of large‐sized cells (FSC‐A > 50K) decreased, middle‐sized MEL cells (blue population) were significantly increased from 14.4% to 57.2%, suggesting these cells were arrested in the middle of differentiation (Figure 3J). These data were further confirmed by MGG staining. We found that compared with the empty vector controls containing large erythroblasts, CAP1 overexpressing group was mainly comprised of smaller (middle‐sized) erythroid cells with purple nuclei (Figure S1C). Taken together, these results demonstrate that CAP1 inhibition favours terminal erythroid differentiation of MEL cells and CAP1 overexpression represses erythroid enucleation.

### 3.4 CAP1 inhibits terminal erythroid differentiation of foetal liver erythroblasts

**Figure 4:**
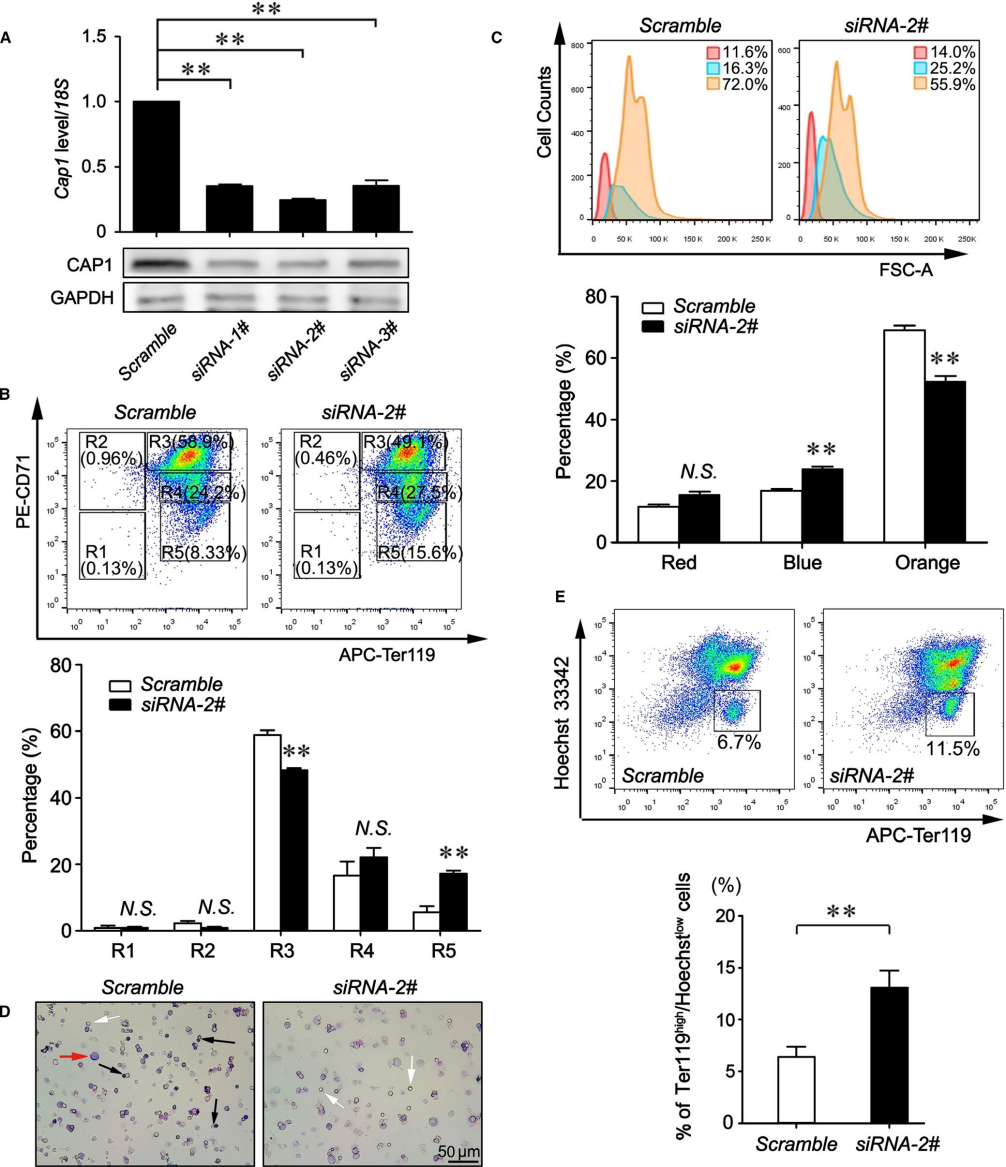
CAP1 inhibits terminal erythroid differentiation of foetal liver cells. A, *Cap1* mRNA and protein levels at day 2 following siRNA knock‐down in primarily isolated Ter119‐ erythroblasts from E12.5 foetal livers. B, Flow cytometry analysis of primary foetal liver erythroblasts at day 2 during differentiation following *Cap1* siRNA knockdown, compared with a scrambled siRNA control. C, The size distribution (FSC‐A) of foetal liver erythroid cells was analysed by flow cytometry at day 2 during differentiation following CAP1 knock‐down. D, MGG staining assays of foetal liver cells at day 2 during differentiation following *Cap1* siRNA knockdown, compared with scrambled siRNA controls. Red arrow indicates erythroblasts, black arrows for enucleating cells and white arrows for reticulocytes without a nucleus. E, Foetal liver cells were stained with APC‐Ter119 and Hoechst 33342 at day 2 during differentiation following *Cap1* siRNA knock‐down. Representative plots of flow cytometry analyses were shown. A, B, C, E, The data are represented as the mean ± SEM (n ≥ 3; **P < .01; *NS*, no significance)

To confirm the negative role of CAP1 in regulating erythroid differentiation, we performed siRNA interfering experiments using in vivo developed erythroblasts from foetal livers. Three siRNAs targeting against *Cap1* mRNA and one scramble control siRNA were designed and transiently transfected into E12.5 foetal liver cells. As shown in Figure 4A, all three *Cap1* siRNAs efficiently blocked *Cap1* expression, with siRNA‐2# showing the highest knock‐down efficiency.

Two days after in vitro culture following siRNA‐2# transfection, we observed a significant increase of late differentiated cells at the R5 stage from 8.3% to 15.6% (Figure 4B). Cell population at R4 stage was also slightly elevated. By contrast, significant declines were detected in the cells at the R3 stage upon CAP1 down‐regulation (Figure 4B). Concomitantly, the proportion of large‐sized cells (above FSC‐A > 50K) decreased from 72.0% to 55.9% (Figure 4C), indicating increased erythroid differentiation upon CAP1 inhibition. In addition, MGG staining showed that CAP1 inhibition speeded up the process of enucleation and cells without nuclei significantly increased (Figure 4D). Consistently, double staining for Ter119 and Hoechst 33342 demonstrated a significant increase of Ter119^high^Hoechst^low^ enucleated reticulocytes from 6.7% to 11.5% upon CAP1 knock‐down (Figure 4E). Notably, we did not observe any obvious alterations in the death of foetal liver erythroblasts in the CAP1 down‐regulated group (Figure S2) rather found a modest decrease in percentage of cells at S‐phase, suggesting a reduced cell proliferation upon CAP1 knock‐down (Figure S3). In conclusion, our results reveal that CAP1 inhibition promotes terminal erythroid differentiation and enucleation of foetal liver cells.

### 3.5 CAP1 inhibits *miR‐144/451*‐mediated terminal erythroid differentiation

**Figure 5:**
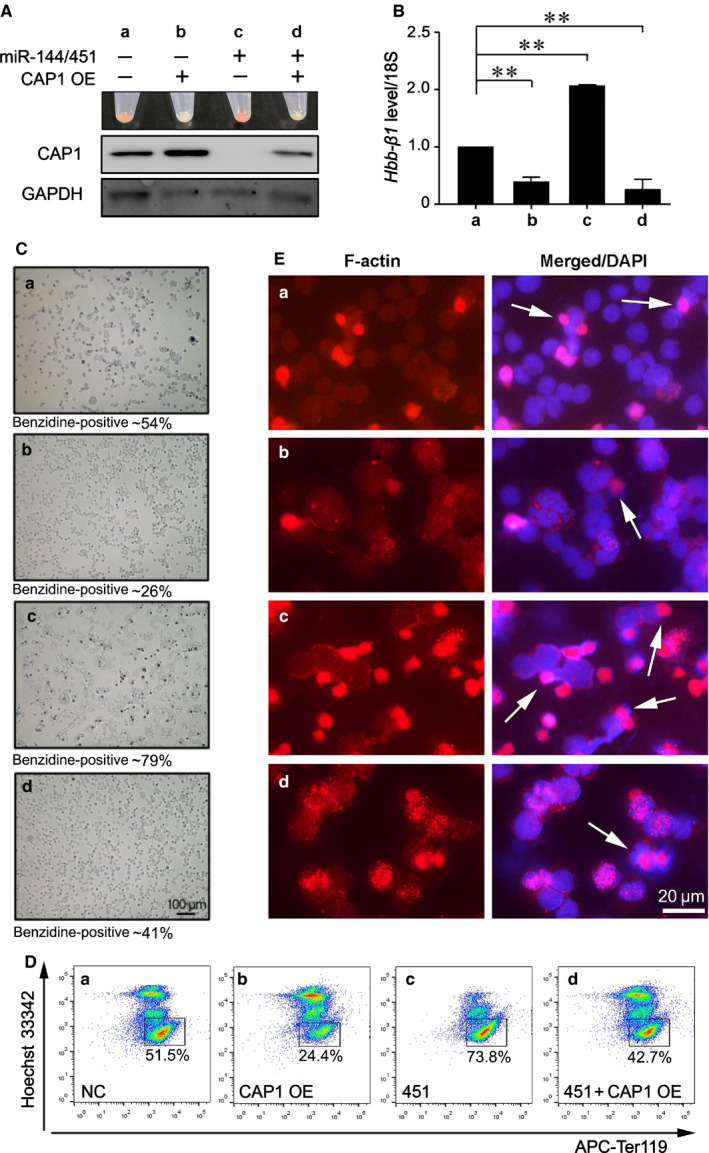
CAP1 inhibits *miR‐144/451*‐mediated terminal erythroid differentiation of MEL cells. A, The gross view of differentiated cell pellets and Western blotting analyses of CAP1 protein levels at day 4 during DMSO‐induced MEL differentiation upon overexpression of *pri‐miR‐144/451* or/and a *Cap1* transgene; (B) The *β‐globin* mRNA levels were analysed by real‐time RT‐PCR assays. C‐E, Benzidine staining (C), flow cytometry analyses with Hoechst 33342/Ter119 double staining (D), and immunofluorescence of CAR (E) of MEL cells at day 4 during DMSO‐induced differentiation. E, The cells were stained with Alexa Fluor 568‐phalloidin. Nuclei were counter‐stained with DAPI. The white arrows indicate CAR in late‐stage erythroblasts. A‐E, Subgroup a: the empty vector control group with no *Cap1* cDNA or *pri‐miR‐144/451* transgene; Subgroup b: MEL cells overexpressing CAP1; Subgroup c: MEL cells with *pri‐miR‐144/451* overexpression; Subgroup d: MEL cells with introduction of both *Cap1* cDNA and *pri‐miR‐144/451* transgene. B, Data are represented as the mean ± SEM (n≥3; **P < .01)

To further determine whether CAP1 inhibition is required for *miR‐144/451*‐mediated erythroid differentiation, we examined erythroid maturation of MEL cells upon co‐expressing CAP1 and *pri‐miR‐144/451* (Figure 5A, and S4). At day 4 after DMSO induction, the pellets from differentiated MEL cells exhibited a much brighter redness in the *miR‐144/451* overexpressing group than that in mock control (Figure 5A), confirming that *miR‐144/451* enhances MEL differentiation. By contrast, the cell pellets displayed a light yellow colour when CAP1 was overexpressed or co‐expressed with both CAP1 and *pri‐miR‐144/451* (Figure 5A and S4). Concomitantly, as shown in the real‐time RT‐PCR assay, *miR‐144/451* significantly promoted *β‐globin* expression which was substantially blocked by CAP1 (Figure 5B). The percentage of benzidine‐positive/dark blue‐stained MEL cells was significantly elevated from 54% to 79% upon *pri‐miR‐144/451* introduction (Figure 5C). This phenomenon disappeared in the presence of CAP1, either alone or co‐overexpressed with *pri‐miR‐144/451* (subgroup b & d, Figure 5C). We thus conclude that CAP1 overexpression directly represses the differentiation of MEL cells induced by *miR‐144/451*.

We next assessed whether CAP1 acts downstream of *miR‐144/451* in regulating erythroid enucleation of MEL cells by flow cytometry with double staining for Ter119 and the DNA dye Hoechst 33342. At day 4 after DMSO‐induced differentiation, we found that the generation of Ter119^high^Hoechst^low^ enucleated MEL cells was significantly repressed by CAP1 overexpression (Figure 5D). By contrast, exogenous *pri‐miR‐144/451* up‐regulated the formation of Ter119^high^Hoechst^low^ cells, but this phenomenon largely diminished when CAP1 transgene was co‐introduced (Figure 5D). Consistent with these findings, we found that *miR‐144/451* facilitated the decrease of cell size during MEL differentiation, analysed by flow cytometry (Figure S5A). The percentage of cells at FSC‐A <30K jumped from 9.5% in the control group to 86.2% (red populations) when *miR‐144/451* was up‐regulated, whereas CAP1 overexpression partially blocked this function of *miR‐144/451* (Figure S5A). We further confirmed with MGG staining assays that the formation of middle‐sized MEL cells with purple nuclei was significantly increased when CAP1 was up‐regulated, whereas exogenous *miR‐144/451* expression promoted the development of small enucleated erythroid cells (Figure S5B). When both ectopic *miR‐144/451* and CAP1 transgenes were introduced (subgroup d), much fewer cells underwent proper enucleation (Figure S5B). These data support our conclusion that CAP1 is a functional downstream target of *miR‐144/451* in erythroid differentiation and enucleation.

Because CAP1 is an actin‐binding protein and may play an important role in maintaining cytoplasmic actin dynamics,^30^ we examined its functional relevance to the formation of CAR during erythroid enucleation. MEL cells were fixed at day 4 after DMSO‐induced differentiation and stained with Alexa Fluor 568‐phalloidin for CAR labelling. Remarkably, *miR‐144/451* promoted the formation of the CAR (red dots in subgroup c), whereas CAP1 alone significantly blocked CAR formation during MEL differentiation (Figure 5E, subgroup b). In addition, few CAR staining was detected when CAP1 was co‐overexpressed with *pri‐miR‐144/451* (Figure 5E, subgroup d), suggesting that CAP1 acts downstream of *miR‐144/451* in erythroid enucleation by regulating F‐actin dynamics.

**Figure 6:**
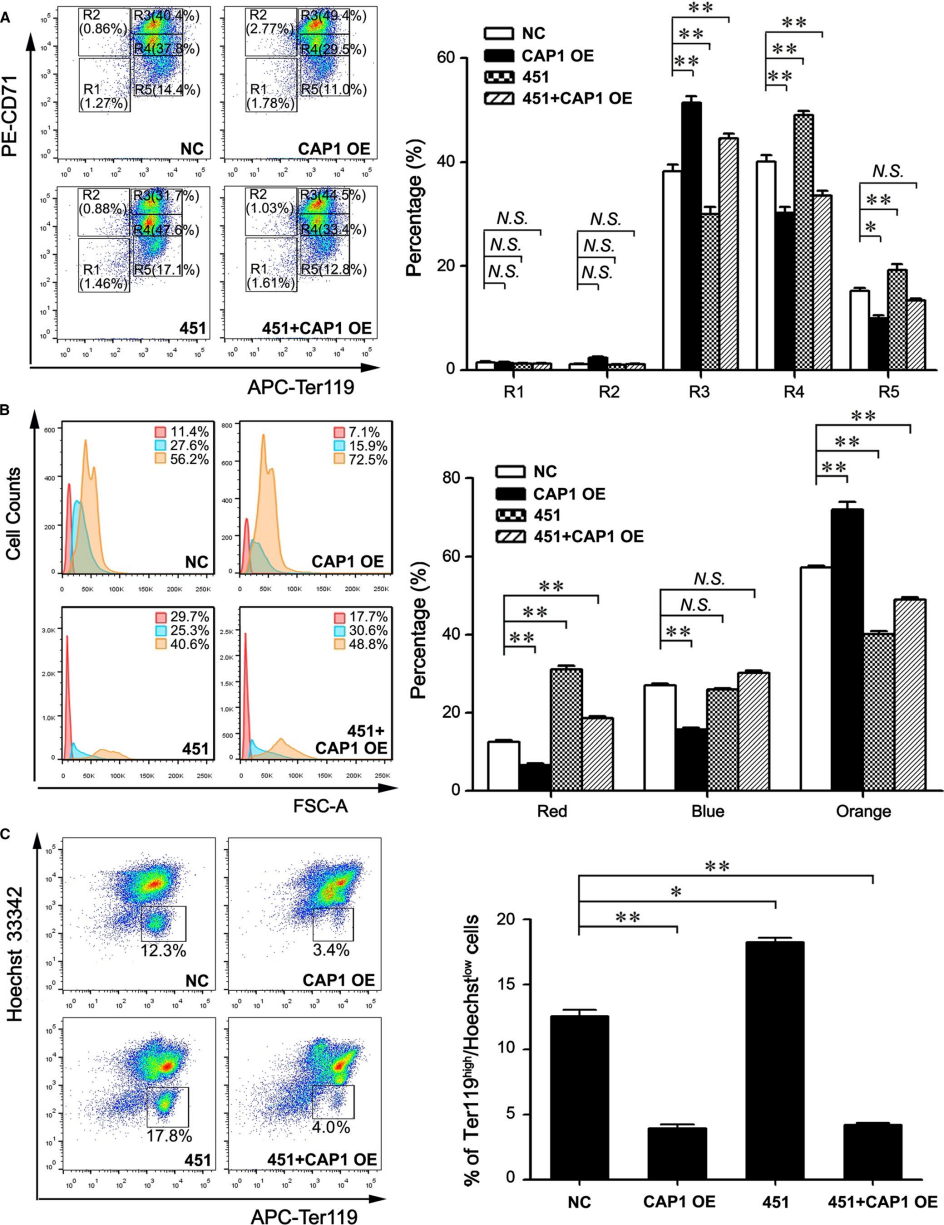
CAP1 inhibits *miR‐144/451*‐mediated differentiation of foetal erythroblasts. A, Flow cytometry analysis of primary foetal liver erythroblasts staining with CD71 and Ter119 at day 2 during differentiation following introduction of *pri‐miR‐144/451* or/and a *Cap1* transgene. B‐C, Flow cytometry analyses of the size distribution (B) as well as Hoechst 33342 and Ter119 co‐staining (C) of primary foetal liver erythroblasts at day 2 during differentiation. A‐C, Subgroup NC: the empty vector control group with no exogenous *Cap1* cDNA or *pri‐miR‐144/451* transgene; Subgroup CAP1 OE: fetal liver cells overexpressing CAP1; Subgroup 451: foetal liver cells with *pri‐miR‐144/451* overexpression; Subgroup 451 + CAP1 OE: foetal liver cells *overexpressing* both *Cap1* cDNA and *pri‐miR‐144/451* transgene. A‐C, The data are represented as the mean ± SEM (n ≥ 3; *P < .05; **P < .01; *NS*, no significance)

To confirm the functional relationship of CAP1 and *miR‐144/451* in regulating physiological erythroid differentiation, we performed similar experiments using CD71+/Ter119− erythroblasts collected from foetal livers. As shown in Figure 6A, two days after in vitro differentiation, we observed a decrease of late differentiated cells at R4 and R5 stages and an elevation of cells at R3 upon CAP1 overexpression (Figure 6A). By contrast, *miR‐144/451* introduction promoted the formation of late differentiated cells at the R4 and R5 stages, but this elevated differentiation by *miR‐144/451* was blocked when CAP1 was concomitantly overexpressed (Figure 6A). In addition, flow cytometry analyses showed that the proportion of large‐sized cells (above FSC‐A >50K) increased from 56.2% to 72.5% upon CAP1 transgene introduction (Figure 6B), indicating a blockage in erythroid differentiation by CAP1. In addition, *pri‐miR‐144/451* promoted the formation of erythroid cells with small size (FSC‐A <30K) from 11.4% to 29.7% (Figure 6B). When CAP1 transgene was co‐introduced, the percentage of these small‐sized cells (below FSC‐A <30K) declined (Figure 6B). Consistent with these findings, the development of Ter119^high^Hoechst^low^ enucleated reticulocytes was significantly blocked by CAP1 when either it was overexpressed alone or co‐expressed with *miR‐144/451* (Figure 6C), as displayed by double staining for Ter119 and Hoechst 33342 in flow cytometry analyses. In summary, our results demonstrate that CAP1 plays a negative role in erythroblast differentiation and enucleation regulated by *miR‐144/451*.

## 4 DISCUSSION

Although a few targets of *miR‐144/451* have been identified, the molecular mechanisms are largely unknown whereby *miR‐144/451* mutations result in phenotypical abnormalities of erythrocytes. Hereby, for the first time, we identified CAP1 as a direct target of *miR‐451* in definitive erythropoiesis. We found that *miR‐144/451* directly inhibited CAP1 expression, but this inhibition was lost when seeding sequences of *miR‐451* in *Cap1* 3′‐UTR was mutated. In addition, our data demonstrated CAP1 acted downstream of *miR‐144/451* to negatively regulate erythroid differentiation and enucleation, thereby supporting CAP1 as a direct and functional target of *miR‐144/451* in mammalian definitive erythropoiesis. Notably, although *miR‐144/451* is transcribed from a bicistronic miRNA genomic locus, CAP1 appears to be a specific target for *miR‐451*. No seed targeting sequence of *miR‐144* was found in *Cap1* mRNA. This may provide an novel explanation for the published findings that *miR‐451* has a greater effect on erythroid maturation than *miR‐144*.^19^

Using both a MEL line and in vivo developed foetal liver‐derived erythroblasts, we found that *miR‐144/451* promoted erythroid differentiation, CAR formation and erythroblast enucleation. Previous studies reported an indispensable role of *miR‐144/451* in erythroid development, however focusing on its regulation of spatial and temporal expression of key genes in protecting red blood cells from oxidative stresses.^20^, ^21^, ^22^, ^23^ Notably, *miR‐144/451^−/−^* mice displayed increased red cell distribution width.^19^ Consistent with this observation, our data demonstrated that *miR‐144/451* acted as an important regulator in the cytoskeleton dynamics and morphologic changes of red blood cells during late stages of erythroid differentiation. Therefore, our study reveals a novel molecular mechanism underlying abnormal morphologic changes of *miR‐144/451^−/−^* erythrocytes and uncovered a previously unrecognized role of *miR‐144/451* in erythroblast enucleation.

Late stage erythroblasts undergo cell cycle exit, chromatin condensation and extrusion of the condensed nuclei *via* an asymmetric cell division.^8^ CAR formation is required for this enucleation process. In immature erythroblasts, F‐actin distributes patchily at cell surface. However, F‐actin bundles become detectable as erythroblasts mature, and finally F‐actin is concentrated to form CAR between the extruding nuclei of erythroblasts and incipient reticulocytes to enable proper enucleation.^48^ We found that, as a direct target of *miR‐451*, CAP1 played a negative role in CAR formation, erythroblast enucleation and terminal erythroid differentiation. CAP1 was gradually down‐regulated along with an up‐regulation of *miR‐144/451* during erythroid differentiation, and its overexpression significantly blocked CAR formation and enucleation driven by *miR‐144/451*. It is plausible that CAP1 directly regulates actin dynamics as a monomeric G‐actin‐binding protein to inhibit polymerization of F‐actin filaments during CAR formation and erythroblast enucleation. Alternatively, CAP1 may enhance cofilin‐mediated F‐actin disassembly and stimulate recycling of cofilin and actin monomers, as reported by a previous study.^49^ In either case, high level expression of CAP1 at early erythroid differentiation may serve as a gatekeeper to prevent erythroblasts from premature enucleation *via* regulating actin dynamics. Notably, in addition to CAP1, a few proteins were identified to regulate erythroblast enucleation through modifying actin dynamics, including RAC1^50^ and RAC2 GTPases,^45^ dynein,^51^ Gelsolin^52^ and mDIA2.^45^ It remains to be determined whether CAP1 interplays with these proteins to precisely regulate the actin dynamics of erythroblasts.

Our study also suggests that CAP1 participates in other aspects of erythroid development. We observed a modest decrease of erythroblasts at S‐phase upon CAP1 inhibition (Figure S3). As terminally differentiated erythrocytes shuts down cell cycles by up‐regulating of p27 and p18 CDK inhibitors,^53^ the reduced proliferation of CAP1 knock‐down cells could be due to increased percentage of mature erythrocytes. CAP1 was previously reported to be a binding partner of adenylyl cyclase and act as a *Ras* effector during nutritional changes in yeasts.^25^, ^28^, ^30^ Interestingly, it is known that RAS signalling plays an important role in terminal erythroid proliferation and differentiation.^11^ We thus could not exclude the possibility that CAP1 is directly involved in cell cycle regulation of erythroblasts. Nevertheless, our study undoubtedly supports a critical role of CAP1 in *miR‐144/451*‐mediated terminal erythroid differentiation and enucleation, thereby contributing to potential clinical therapy in the field of blood transfusion.

## CONFLICT OF INTEREST

The authors confirm that there are no conflicts of interest.

## AUTHOR CONTRIBUTIONS

**Xiaoli Huang:** Conceptualization (equal); Data curation (equal); Formal analysis (equal); Methodology (equal); Software (equal); Validation (equal). **Ruihua Chao:** Data curation (equal); Investigation (equal); Methodology (equal); Validation (equal). **Yanyang Zhang:** Data curation (equal); Methodology (equal). **Pengxiang Wang:** Data curation (equal); Formal analysis (equal); Methodology (equal). **Xueping Gong:** Data curation (equal); Methodology (equal). **Dongli Liang:** Data curation (equal); Formal analysis (equal); Methodology (equal); Project administration (equal); Supervision (equal); Validation (equal); Writing‐original draft (equal); Writing‐review & editing (equal). **Yuan Wang:** Conceptualization (equal); Formal analysis (equal); Funding acquisition (equal); Investigation (equal); Methodology (equal); Project administration (equal); Resources (equal); Supervision (equal); Validation (equal); Visualization (equal); Writing‐original draft (equal); Writing‐review & editing (equal).

## Supporting information

Fig S1‐S5

Table S1

## Data Availability

The data that supports the findings of this study are available in the Supporting information of this article.
