# Supplementary material for: CAP1, a target of miR‐144/451, negatively regulates erythroid differentiation and enucleation
Source: J Cell Mol Med. 2021 Jan 26;25(5):2377–89. doi: 10.1111/jcmm.16067 (PMC7933962; doi:10.1111/jcmm.16067)
Supplement: Supplementary file 1 — Fig S1‐S5 [file JCMM-25-2377-s001.docx]

**
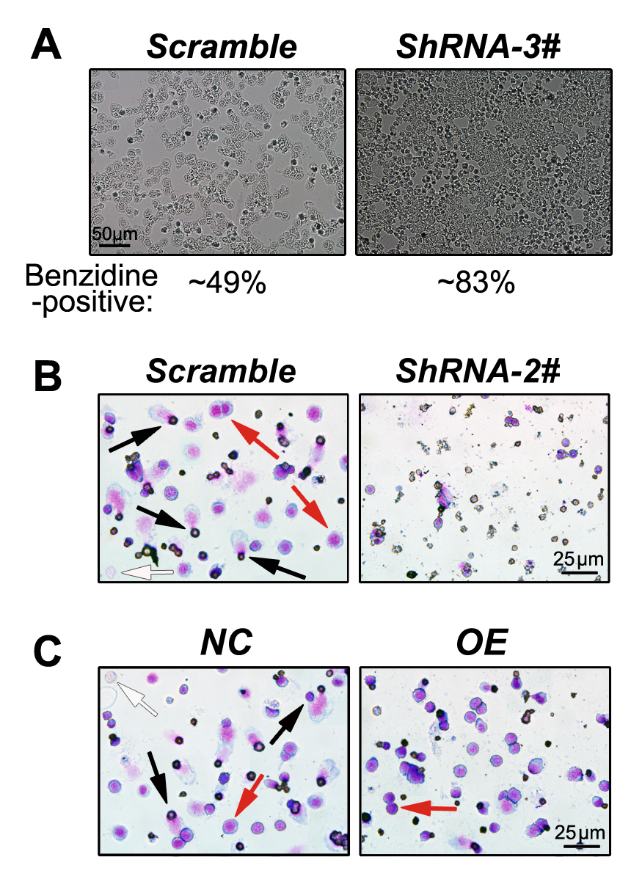
**

**Supplemental Figure S1.** **CAP1 inhibits erythroid differentiation and enucleation of MEL.** (A) Benzidine staining of MEL cells at day 4 post DMSO-induced differentiation upon CAP1 knockdown. (B-C) MGG staining of MEL cells at day 4 post DMSO-induced differentiation upon CAP1 knockdown (B) or overexpression (C). Red arrows indicate erythroblasts, black arrows for enucleating cells, and white arrows for reticulocytes without a nucleus.

**
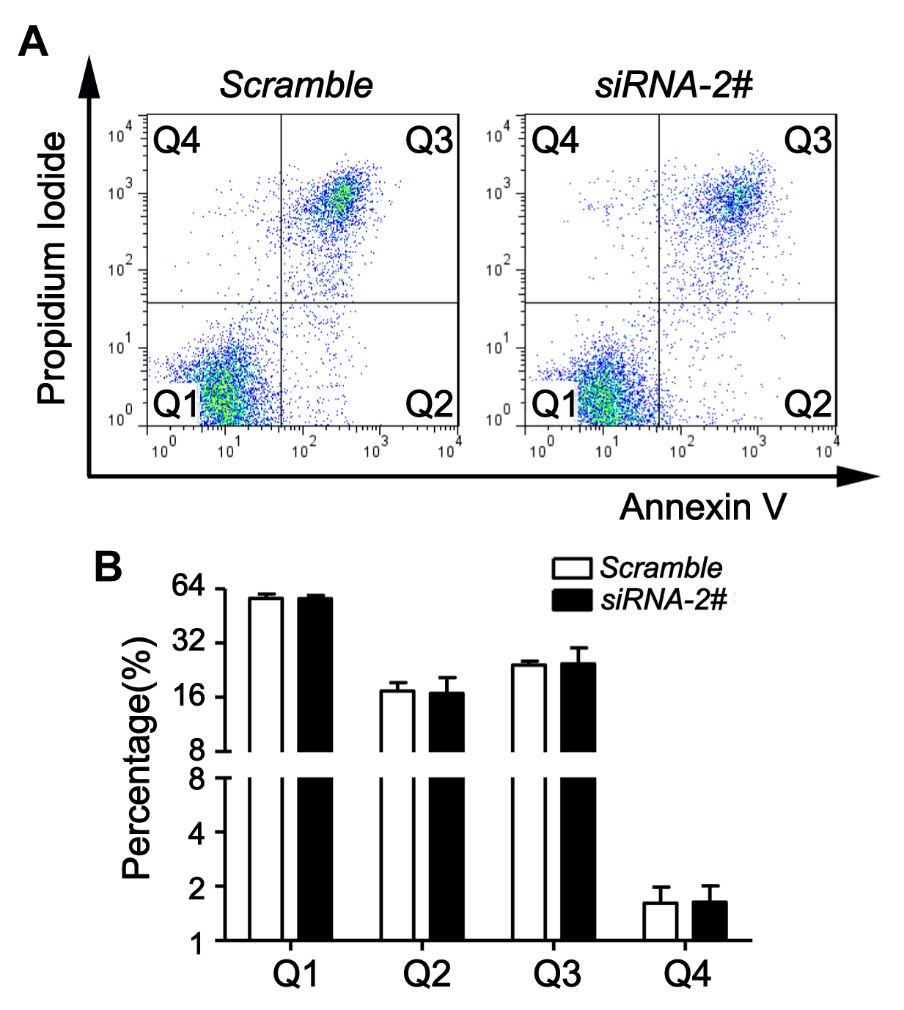
**

**Supplemental Figure S2.** **Knockdown of CAP1 in fetal liver erythroblasts did not cause apoptosis.** (A) Apoptosis of erythroid cells was analyzed by flow cytometry at day 2 during differentiation post *Cap1* siRNA knockdown in E12.5 fetal liver erythroblasts. (B) Quantitative analyses of flow cytometric data from (A). The data are represented as the mean ± SEM from two biological replicates.

**
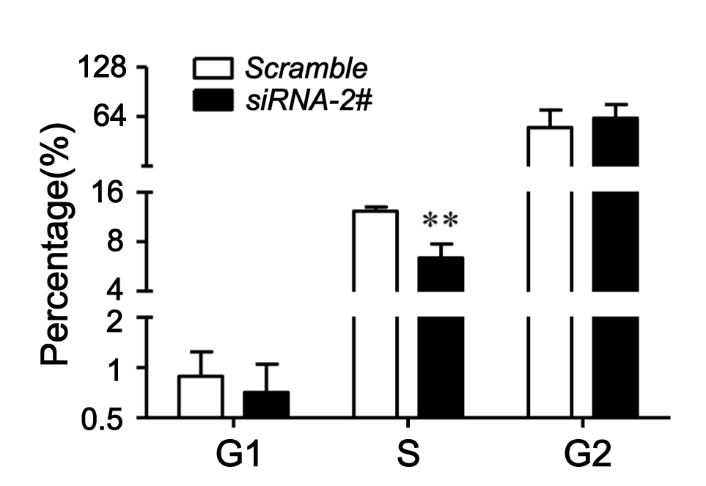
**

**Supplemental Figure S3. Knockdown of CAP1 in fetal liver erythroblasts leads to the decreased cell percentage in S phase.** Cell cycle analysis of erythroid cells at day 2 during differentiation post *Cap1* siRNA knockdown in E12.5 fetal liver erythroblasts. The data are represented as the mean ± SEM (n≥3, ***P* < 0.01).


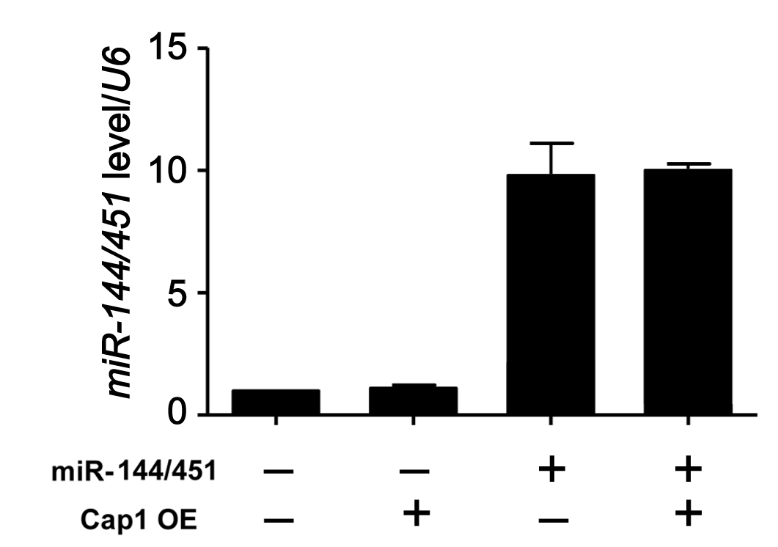


**Supplemental Figure S4. Expression of miR-144/451 at day 4 during DMSO-induced MEL differentiation.**


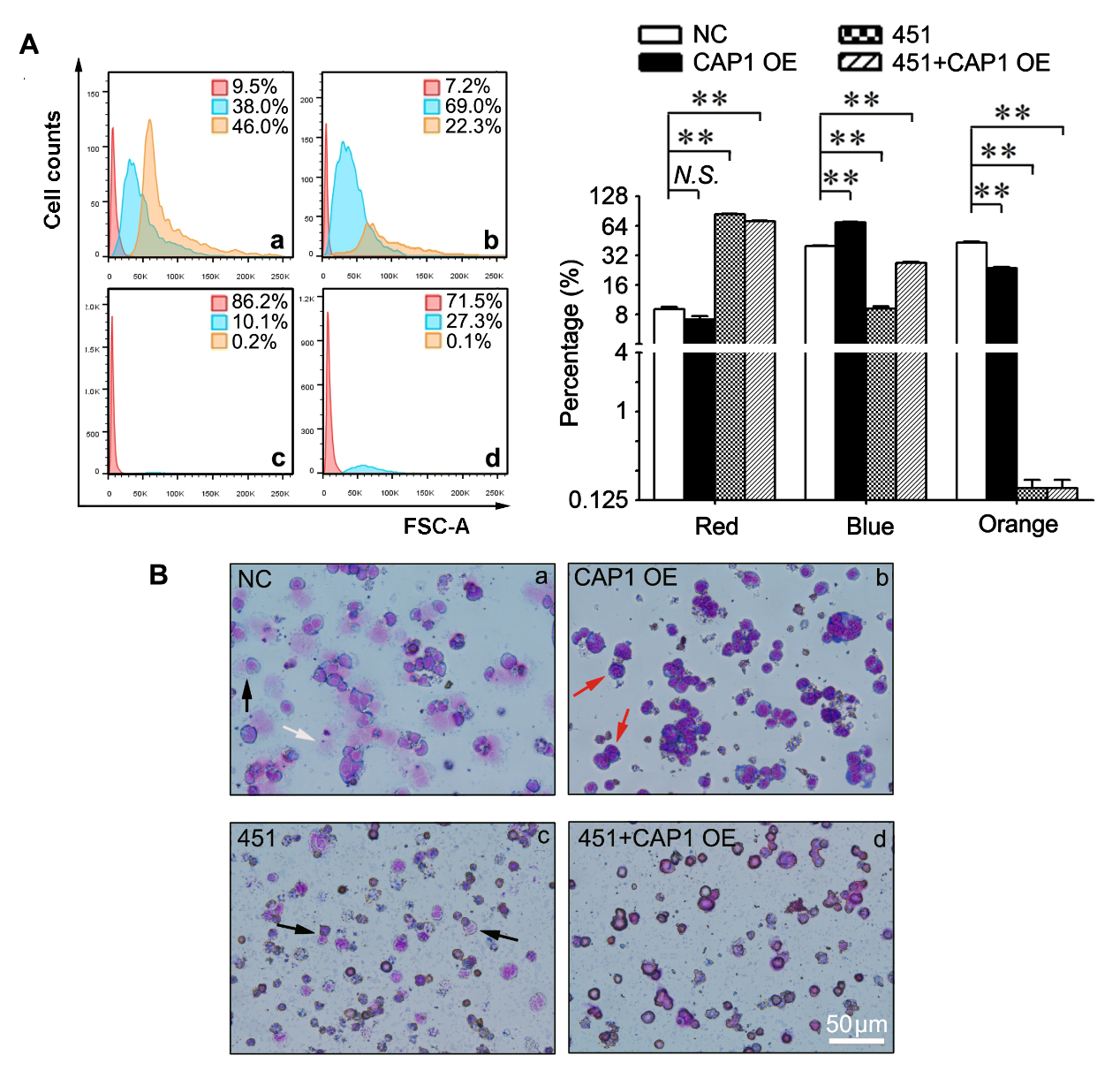


**Supplemental Figure S5. CAP1 inhibits *miR-144/451*-mediated terminal erythroid differentiation and enucleation of MEL cells.** (A-B) The size distribution (FSC-A) (A) and MGG staining (B) of MEL cells at day 4 during DMSO-induced differentiation. Subgroup a: the empty vector control group with no exogenous *Cap1* cDNA or *pri-miR-144/451* introduction; Subgroup b: MEL cells overexpressing CAP1; Subgroup c: fetal liver cells with *pri-miR-144/451* overexpression; Subgroup d: fetal liver cells with introduction of both *Cap1* cDNA and *pri-miR-144/451* transgene. Red arrow indicates erythroblasts, black arrows for enucleating cells, and white arrows for reticulocytes without a nucleus. The data are represented as the mean ± SEM (n≥3; ***P* < 0.01; *N.S.*, no significance).
