# Supplementary material for: CAP1, a target of miR‐144/451, negatively regulates erythroid differentiation and enucleation
Source: J Cell Mol Med. 2021 Jan 26;25(5):2377–89. doi: 10.1111/jcmm.16067 (PMC7933962; doi:10.1111/jcmm.16067)
Supplement: Supplementary file 2 — Table S1 [file JCMM-25-2377-s002.docx]

| Methods | Primer Name | Primer Sequence |
| --- | --- | --- |
| Real-time PCR | *Cap1* Forward | TCACACATGCCCTGAAACAT |
|  | *Cap1* Reverse | GTTTGGGGTTTAGGTGCAGA |
|  | *Hba (α1+α2)* Forward | CACCACCAAGACCTACTTCC |
|  | *Hba (α1+α2)* Reverse | CAGTGGCTCAGGAGCTTGA |
|  | *Hbb (β1+ β2)* Forward | ACCCAGCGGTACTTTGATAGC |
|  | *Hbb (β1+ β2)* Reverse | CCTGAAGTTCTCAGGATCCACAT |
|  | *18S* Forward | AAACGGCTACCACATCCAAG |
|  | *18S* Reverse | CCTCCAATGGATCCTCGTTA |
|  | *Pri-miR-144/451* Forward | ATGCAGAAGTACACGGGCTC |
|  | *Pri-miR-144/451* Reverse | TTCCTCGCCATTCCCAAGTG |
|  | *U6* Forward | CGCTTCGGCAGCACATATA |
|  | *U6* Reverse | CTTCACGAATTTGCGTGTCA |
| Luciferase reporter assay | *Cap1* 3'-UTR Forward | GAACTCAGGAAGAGCAGTCA |
|  | *Cap1* 3'-UTR Reverse | GGATGGAATGCAGATGAAGG |
| Overexpression | *Cap1* Forward | CAGATTCTCTCTCCGGTGGC |
|  | *Cap1* Reverse | CTCAGGTAATGGGCAGAAGTCA |
|  | *Pri-miR-144/451* Forward | AGTGGAGGAGCAGAGGTAGG |
|  | *Pri-miR-144/451* Reverse | AGTTTGTGGGAGCAGCAAGA |
| shRNA knockdown | Scramble shRNA | GTCTAGCTAACAGTCGTTCCTACGGCTT |
|  | *Cap1* shRNA-1# | GCTACAGCTTCTCAGTGCCAGCAGCCAGC |
|  | *Cap1* shRNA-2# | GTAGATAACTGTAAGAAGCTTGGCCTGG |
|  | *Cap1* shRNA-3# | GCTTGTCACTCGGCAGCTTAGGGCCTCTG |
| siRNA interfering (GenePharma biotechnology company ) | *Cap1*-1# sense | GUAAGCUCUGACCCAGUCCACA |
|  | *Cap1*-1# antisense | UGUGGACUGGGUCAGAGCUUAC |
|  | *Cap1*-2# sense | AGAGCAGAAAGGUAGAGGAAAU |
|  | *Cap1*-2# antisense | AUUUCCUCUACCUUUCUGCUCU |
|  | *Cap1*-3# sense | GCUGCGCUGAUGCCGAGAAGCU |
|  | *Cap1*-3# antisense | AGCUUCUCGGCAUCAGCGCAGC |
